# St. Louis Medico Chirurgical Society

**Published:** 1879-12

**Authors:** 


### ST. LOUIS MEDICO CHIRURGICAL SOCIETY.

*Summer Diarrhœas of Children.*—Dr. Montgomery: Some three years ago I published an article in which I gave my ideas rather fully with regard to the summer affections of children, and was very much pleased, the other day, by seeing some high authorities agree with me in one of the chief points I made, viz: that those cases reported in our mortuary statistics as cholera infantum, are cases of inflammatory diarrhœa—entero-colitis, certainly a very malignant disease, and very hard to treat, and so insiduous in its onset that many children die from it. We have quite a number of diseases in children here, occurring in the summer time; for instance, simple mucous or catarrhal diarrhœa, entero-colitis, cholera infantum and dysentery. The most fatal of these is entero-colitis. In simple catarrhal diarrhœa in children, very simple remedies, warm fomentations to the bowels, particular attention to the diet, mucilaginous and wholesome kinds of drinks and food, will generally effect a cure without much trouble. I would here say it is very important to scarify those children as soon as you find the gums are swollen or enlarged, and to do it again and again. At one time I was very much prejudiced against much scarifying, but I have seen so many cases in which it has been of benefit, that I have changed my opinion. I have sometimes scarified a child as often as six or seven times, with excellent effect. I have also found, in my experience, an excellent remedy in bromide of potassium. In teething cases, where the children are irritable, it is a most useful remedy. I give two grains to a child a year old, every three hours or so; and I also very often give a little antacid, such as bicarbonate of soda, etc., which will sometimes alleviate those cases.

I look upon entero-colitis as an inflammatory disease—decidedly so. When a physician is called to a case of that kind, he will generally find the child feverish, the fever being either intermittent or remittent in type. There will be some portions of the twenty-four hours in which the child will be very hot and dry, and it will have a great inclination to throw up. The bowels will be exceedingly irritable, and the stools will be very varied in character, sometimes putrid and mixed up with slimy mucus; and if the case is aggravated, a little blood will occasionally appear. The child will become thin, and wish to lie on its face, will have no appetite, but will drink incessantly. My favorite drink for them is barley water. A little ice-water, or the white of eggs beaten up in water, or a solution of gelatine, are all very good. But in those cases, I believe very much in leeching the hypogastrium, followed by hot fomentations, with which I generally mix a little mustard. If the evacuations from the bowels are of a clay-bank color, or turbid, or indicate torpidity of the liver, I give a fractional dose or two of calomel, until the character of the stool is changed. I must say that in those cases of entero-colitis in which there is great irritability both of the stomach and bowels, I have great faith in small doses of mercurials.

They may often be given in conjunction with prepared chalk and a little Dover's powder. Ipecac is a remedy which has a most happy effect in those cases where there is an inflammatory action of the mucous membrane of the bowels, and has also a favorable action on the liver. I believe all authorities agree that in those cases, when fatal, the mucous membrane of the alimentary canal will be changed into softened and diseased tissue, showing that there has been a very serious organic disease going on, and that the inflammation in the first stages has been severe, has led to structural lesion of the intestines; and, then, when that has taken place, any remedy is very apt to fail. In those cases where I think there have been pathological changes of that kind, I have often tried other remedies, as the liquor ferri pernitratis, one or two drops four or five times a day, in a little water. Where the child is of a really scrofulous constitution, after mercurial doses I have often given iodide of iron with cod-liver oil in a very weak emulsion, and it agrees with them very well. Bismuth is a very popular remedy, but I have not such faith in it as most have. It is an almost universal remedy in sub acute cases of diarrhœa, but I have not much confidence in it. I will remark that generally when it is given it should not be given in milk. When I prescribe bismuth at all, I give it with gum arabic and a little sugar dissolved in water. Sometimes it has a very good effect in lessening watery operations. One thing in my experience—I may be wrong, but I have a very strong prejudice against using astringents in those cases of diarrhœa. In looking over the prescriptions in drug-stores, you will find that there has been for years gone by a very prevalent custom of giving astringents, such as tincture of catechu, kino, laudanum and paragoric. I believe that head troubles are apt to arise under their use. I am really afraid to give those astringents, except in cases which have become chronic.

With regard to cholera infantum, I think it is a great mistake in registering so many of those cases as such. Cholera infantum in children is very like cholera morbus in the adult—they generally get well. I call to mind a great many cases of cholera infantum where they were apparently hopeless, where I was about to give them up, where, when a cup of fluid was given them, they would drink the contents with the utmost greediness, and almost try to eat the cup; but even those cases have generally got well by simply using mucilaginous drinks, poultices to the abdomen, and so forth. In those cases of cholera infantum, the stools are generally like those in cholera morbus—almost colorless, like rice-water or chicken soup. If you try and check them by giving astringents, you will almost certainly seriously injure your patient. I give a little mercurial to act on the liver, then make hot applications to the abdomen; counter-irritation there is very important, both in cholera infantum and entero-colitis.

I do not see how it is so many patients should be set down in the mortality reports as dying from cholera infantum, because the affections in those cases are so very distinct.

Dr. Gregory: How do you like saline aperients in conditions similar to those you refer to?

Dr. Montgomery: In a *strong* child and in the early stages of entero-colitis, I have great faith in giving a little Rochelle salts.

Dr. Gregory: How do you like sulphate of magnesia?

Dr. Montgomery: In dysentery, in a grown person, I have great faith in it.

Dr. Gregory: Do you combine opiates with the salines?

Dr. Montgomery: I prefer to give the saline alone, but I occasionally give a little Dover's powder.

Dr. Gregory: Has it been your observation, Dr. Montgomery, that so-called cases of cholera infantum are often complicated with head trouble?

Dr. Montgomery: Yes, sir.

Dr. Gregory: And the possibility is that the fatal cases resulting from head symptoms are not simple cholera infantum?

Dr. Montgomery: Yes; and that is the reason I alluded to the use of bromide of potassium in those cases.

Dr. Gregory: Do you find that the application of leeches does good in those cases?

Dr. Montgomery: Yes, sir.

Dr. Gregory: I would like your opinion with regard to a very commonly spoken of disorder, but about which I have had some doubts with regard to its nature, namely: tubercular meningitis—a very common name in mortality reports—whether tubercular meningitis, so-called, is not often a simple meningitis?

Dr. Montgomery: Yes, I think it is.

Dr. Kingsley: But at the same time you will find a great many cases of meningitis in which you can find no tubercle at all. But in genuine cases of tubercular meningitis you will very generally find tubercle in some portion of the body, in the lungs, or elsewhere. Sometimes the presence of tubercle can be ascertained by simply making an examination of the eye. I remember a post-mortem that was made in the hospital of Paris, where one of the *internes* made an examination, removing the eye and examining the humors of the eye, and made an exhibition of the tubercular matter which he found there. They not unfrequently made use of examination of the eye in their diagnosis. It was only a very minute granular matter; they would be unable to see it with the naked eye.

Dr. Carson: I would like to ask some of the experienced gentlemen present if they do not find that most, or many of the diarrhœas of children are the result of indigestion, and if they, again, do not find it a very difficult matter to find proper nourishment for children?

Dr. Kingsley: I think that nearly all cases are due to indigestion. I know Jacobi makes this statement, that forty per cent. of deaths in the first year of life are from derangement of the digestive organs. Further: I know Jacobi makes the statement also, that it is a fallacy to believe that the second summer with children is the dangerous season; that in reality it is the first. You may have noticed in the statistics of last week that seventy-seven children died under one year of age, and that only a hundred in all died. Jacobi says that in the first year of life the fatal diseases are chiefly of the alimentary canal, and in the second, pulmonary diseases. A vast majority of children are eating things they should not eat; mothers frequently tell us that they give various substances, even fruits. Then, on the other hand, they are often fed too frequently during the day. A mother came to me to-day who wanted to nurse the child every half hour—a child about two months old. As a matter of course, it takes some little time to digest even milk, and milk that is in the stomach should be digested before any more is given.

Dr. Spencer: Negro women, as you know, put a lump of fat beef in the child's mouth as soon as it is born.

Dr. Gregory: What is your opinion, Dr. Montgomery, of lime-water?

Dr. Montgomery: I think it is very excellent, although I do not like to give much of it, because I think that after a while it has a depressing effect on the digestive organs. But where there is much acidity of the stomach a little lime-water, with good sweet milk, is an excellent thing. My favorite remedy is bicarbonate of soda. I forgot to mention that in many cases of entero colitis, the child has very large stools; and where these are present, I find a very excellent remedy in pepsin and nitro-hydrochloric acid, with a little syrup and water, to be given immediately after it takes its food.

Dr. Gregory: Do you use pepsin or lactopeptin?

Dr. Montgomery: I have used both; but my favorite recipe is the pepsin and nitro-hydrochloric acid.

Dr. Kingsley: For those troubles, entero-colitis, Smith, in one of his reports to the Medical Society of New York, not published in his book, gives this prescription :

℞.—Bismuthi subnitratis..................ʒii
Lactopeptini.........................ʒiss
Tr. opii deodoratæ................gtt. xvi
Syrupi simplicis......................℥ss
Misturæ cretæ.........................℥iss
S.—Teaspoonful to a child one year old.

Dr. Montgomery: I do not believe in combining bismuth with an acid.

Dr. Kingsley: Jacobi gives this prescription: 1 gr. subnitrate bismuth, 2 grs. prepared chalk, and about one-third grain Dover's powder; one powder every three or four hours.

Dr. Gregory: There is a point in the remarks which I think an interesting one; that in regard to the use of minute doses of mercurials. There is certainly a good deal of difference of opinion in the profession with regard to that point. My preceptor taught me thirty years ago to give mercurials every four hours, with sometimes a little soda, sometimes a little chalk, and I have followed it up to the present day without any reason for changing, and yet I am frequently brought into consultation with doctors and I find they have no faith in the thing The Doctor's experience seems to have accorded fully with my observations, and I have been amazed, from time to time, during my professional life, at the wonderful effect of minute mercurials. I put a little antacid with it and throw it dry on the tongue. Vomiting stops by its use, the diarrhœa is arrested, the dejections are all changed in a little while, and the general condition is ameliorated rapidly. This, with a little chalk mixture, will stop a very large proportion of disorders of digestion in children. Yet, I have met with excellent physicians who never use it at all.

Dr. Kingsley: I have found that sometimes minute doses of calomel will arrest vomiting and prevent decomposition in the alimentary canal, in consequence of the calomel being converted into a sublimate. I think salicylic acid is very good in those cases where the discharges are very foul and disagreeable.

Dr. G. A. Moses: I was glad to hear Dr. Montgomery's remarks, based upon a long, extensive, and successful experience, and because his views accord with and confirm my own. Like him, I believe that a large number of cases called cholera infantum are not cholera infantum at all. The majority of such cases will get well by themselves. When they do not, it is because of some complication, or they are in an inflammarory condition and result in entero-colitis or gastro-enteritis or brain trouble. In regard to the brain trouble, I consider cholera infantum a disease of the nervous system—of the nerves of animal life. Where the irritation is very great, and the discharges very large and rapidly following each other, we have a reflex condition affecting the brain, resulting in convulsions, with perhaps œdema secondarily, resulting from anæmia—the so-called "spurious hydrocephalus." Only a few days ago I had an example of the spontaneous cure of cholera infantum. The child was exhausted, vomiting everything, even a teaspoonful of cold water. I ordered a mixture of magnesia to be given every fifteen or twenty minutes, with the egg drink that Dr. Montgomery speaks of. When I got home, about ten hours afterward, I found a message from the mother that she had lost the prescription, but the child had stopped vomiting and purging, simply on the use of a little iced egg-water, and the next morning it was well. I think that would be the history of many such cases. But where purging is frequent, and especially where the operations are very thin, white or slightly yellow, exhibiting a condition of infiltration of the mucous tract, small doses of mercurials, from one-sixth to one-tenth of a grain of the mild chloride, every hour, are most beneficial. As to the use of opiates and astringents, I think they are absolutely pernicious, and result in producing the sequelæ of the disease, which we have to fear. Oftentimes there is intense collapse, then I find a little old brandy the best stimulant. Bromide of potassium answers oftentimes very well in allaying the nervous irritation, without the use of opiates.

Dr. Gregory: Has it not occurred to you that when you see a case of cholera infantum, that when you have made up your mind that the disease will cure itself, that if you had seen the case before the cholera infantum had declared itself, that you would have given some dose that would have caused diarrhœa? My patients sometimes say, after I give them a dose, that they have had a fearful purging.

Dr. Moses: Wherever a fever manifests itself, I consider mercurials a necessity. In regard to vomiting, I have found that the old spice poultice is excellent; if you cannot get that, hot fomentations will give very prompt relief. Sometimes, mixed with this, a little quinine or cinchonidia, has a very happy effect. In diseases of an inflammatory character, my experience coincides with that of Dr. Montgomery. I am satisfied that salines do not answer the same purpose as mercurials. The use of astringents in diarrhœal affections in children, after the acute stage has passed away, will often be necessary. There will simply result an irritation, debility of the blood vessels, where there is frequeht peristaltic action on the one hand, and an increased discharge of serosity on the other. For prolonged discharges of blood, an occasional opiate, combined with an astringent, acts very beneficially. For this purpose I find liq. ferri pernitratis acts excellently. My chief objections to fomentations is the increased heat they produce. The diseases to which we now refer are hot-weather diseases, and I do not consider an increase of temperature desirable. Summer diarrhœa is considered by some to be due to bacteria, which play so important a figure in surgery, and bacteriacides have been recommended—quinine, salicylic acid, and very minute doses of corrosive sublimate.—*St. Louis Courier of Medicine.*

